# The Effect of EMDR and CBT on Low Self-esteem in a General Psychiatric Population: A Randomized Controlled Trial

**DOI:** 10.3389/fpsyg.2017.01910

**Published:** 2017-11-08

**Authors:** Brecht T. Griffioen, Anna A. van der Vegt, Izaäk W. de Groot, Ad de Jongh

**Affiliations:** ^1^Dimence, Zwolle, Netherlands; ^2^Behavioural Sciences and Social Dentistry, University of Amsterdam, Vrije Universiteit Amsterdam, Amsterdam, Netherlands; ^3^School of Health Sciences, University of Salford, Manchester, United Kingdom; ^4^Institute of Health and Society, University of Worcester, Worcester, United Kingdom

**Keywords:** self-esteem, EMDR, CBT, psychiatric population, randomized controlled trial

## Abstract

Although low self-esteem has been found to be an important factor in the development and maintenance of psychopathology, surprisingly little is known about its treatment. This study investigated the effectiveness of Eye Movement Desensitization and Reprocessing (EMDR) therapy and Cognitive Behavioural Therapy (CBT), regarding their capacities in enhancing self-esteem in a general psychiatric secondary health care population. A randomized controlled trial with two parallel groups was used. Participants were randomly allocated to either 10 weekly sessions of EMDR (*n* = 15) or CBT (*n* = 15). They were assessed pre-treatment, after each session, post treatment and at 3 months follow-up on self-esteem (Rosenberg Self-esteem Scale and Credibility of Core Beliefs), psychological symptoms (Brief Symptom Inventory), social anxiety, and social interaction (Inventory of Interpersonal Situations) (IIS). The data were analyzed using repeated measures ANOVA for the complete cases (*n* = 19) and intention-to-treat (*n* = 30) to examine differences over time and between conditions. Both groups, EMDR as well as CBT, showed significant improvements on self-esteem, increasing two standard deviations on the main parameter (RSES). Furthermore, the results showed significant reductions in general psychiatric symptoms. The effects were maintained at 3 months follow-up. No between-group differences could be detected. Although the small sample requires to exercise caution in the interpretation of the findings, the results suggest that, when offering an adequate number of sessions, both EMDR and CBT have the potential to be effective treatments for patients with low self-esteem and a wide range of comorbid psychiatric conditions. This study was registered at www.trialregister.nl with identifier NTR4611.

## Introduction

Self-esteem has been defined as a person's overall evaluation of his or her own worth (Hewitt, 2009). Low self-esteem is involved in a wide range of psychiatric conditions, including depression (Brown et al., 1990), anxiety disorders (Sowislo and Orth, 2013), personality disorders (Lynum et al., 2008) obsessive compulsive disorder (Ehntholt et al., 1999), eating disorders (Gual et al., 2002), chronic pain (Soares and Grossi, 2000), substance abuse (Silverstone and Salsali, 2003), and psychosis (Barrowclough et al., 2003). Research suggests that low self-esteem increases the susceptibility for development of these psychiatric disorders, and that, in turn, the presence of a psychiatric condition negatively influences someone's self-esteem (Silverstone and Salsali, 2003). There is also considerable evidence to support the notion that in general self-esteem is a reliable predictor of treatment outcome, in that higher initial self-esteem is significantly associated with better treatment outcomes (Johnson et al., 2000; Parker et al., 2013). It can be concluded that low self-esteem is an important factor in relation to psychiatric disorders in general.

Over the past several years a variety of therapeutic interventions has been developed for changing low self-esteem, predominantly with a cognitive behavioral background. These interventions mostly aim at changing core beliefs underlying patients' low self-esteem (Padesky, 1994; Beck, 1995; Fennell, 1999). Several case studies (Fennell, 1998; McManus et al., 2009) and clinical trials (Rigby and Waite, 2006; Waite et al., 2012) suggest that these interventions are effective in enhancing self-esteem. However, only a few studies have compared Cognitive Behavioural Therapy (CBT) to an active or passive control group using a randomized controlled trial. One study examined the effectiveness of CBT on improving implicit and explicit self-esteem in patients with a social anxiety disorder, comparing this to psychodynamic therapy using 25 sessions (Ritter et al., 2013), and found a positive treatment effect for both treatments. Another study found a positive effect of CBT being significantly more effective in changing self-esteem in comparison to a waitlist control condition (Waite et al., 2012). Some studies have also addressed the effectiveness of group CBT on individuals' self-esteem, mostly using protocols designed by Fennell (1998), showing significantly positive treatment effects, including a reduction of symptoms of depression and anxiety (Rigby and Waite, 2006; Morton et al., 2012; Pack and Condren, 2014). Hence, research thus far found support for the effectiveness of CBT for individuals suffering from low self-esteem.

It is an observation in clinical practice that when treating low self-esteem in patients with psychiatric comorbidities or more severe symptoms of psychiatric conditions, the application of cognitive interventions may not always be sufficient to effectively change patient's core beliefs. Patients frequently report that they still “feel” bad about themselves, albeit rationally believing that their core beliefs are not true (Young et al., 2002; Sanders and Ten Broeke, 2011). This suggests that a treatment that would intervene in a different manner, perhaps on a more affective level, and make patients actually “feel” more worthy, could be more effective, or at least be an additional tool for enhancing self-esteem.

Eye Movement Desensitization and Reprocessing (EMDR) therapy is considered to be a treatment method that intervenes on a more affective level (Shapiro, 2001). EMDR therapy is a protocolized psychotherapeutic treatment that is used to treat symptoms caused by distressing and unprocessed life events through reducing the vividness and disturbance of the memories of such events (Shapiro, 2007; Solomon and Shapiro, 2008). Although EMDR is mainly used for treating posttraumatic stress disorder (PTSD), it has been argued that EMDR therapy might also be an effective therapy for changing low self-esteem (De Jongh et al., 2010). Assuming that core beliefs underlying the low self-esteem developed as a consequence of subsequent learning experiences, EMDR may be used to reprocess emotionally charged memories that the patient considers to be “evidence” for his or her core belief (De Jongh et al., 2010). According to this case conceptualization, processing these memories using EMDR would make it possible to re-evaluate the present meaning of those experiences, thereby positively influencing their self-esteem.

Several case studies have shown a positive effect of EMDR on low self-esteem (Dziegielewski and Wolfe, 2000; Shapiro, 2001; Maxwell, 2003; Sanders and Ten Broeke, 2011). The results of a randomized controlled trial among 26 adolescents with self-esteem and behavioral problems showed that EMDR was effective in enhancing their self-esteem (Wanders et al., 2008). The researchers used four sessions EMDR therapy and compared this to four sessions of CBT, which contained strategies to teach children practical skills, to identify negative feelings and unhelpful thoughts, to replace these with more positive thoughts and to face and overcome their problems and challenges. Although both therapies where found to be effective, EMDR resulted in significantly more behavioral changes than CBT. Recently, Staring et al. (2016) used a randomized controlled trial with a crossover design among 47 adults with anxiety disorders to compare six sessions EMDR therapy with an equal number of sessions Competitive Memory Training (COMET) that aims to activate positive representations for enhancing self-esteem. They found that EMDR improved self-esteem, but they found a significantly stronger effect of COMET compared to EMDR therapy. Thus, the few studies that investigated the effectiveness of EMDR applied on self-esteem have so far shown mixed results. There are some explanations for these contradicting findings. First, until now, only a few sessions (4–6) of EMDR therapy have been used. It is conceivable that for changing individuals' long existing negative core beliefs, a wide array of memories would have to be targeted, “proving” that the person is bad or worthless. Furthermore, it could be argued that in the study of Staring et al. (2016) the memories that were targeted with EMDR, and that were deemed to contribute to patients' low self-esteem, could have been relatively low in emotional charge and, consequently, less sensitive to EMDR (Littel et al., 2017). Therefore, it could be hypothesized that especially patients with severe pathology and multiple diagnoses, associated with lower self-esteem (Silverstone and Salsali, 2003), might have memories underlying their low self-esteem with higher emotional charge, making them more likely to benefit from EMDR therapy.

The purpose of the current study was to test the effectiveness of EMDR therapy in adults with low self-esteem in a secondary mental health care population, by comparing it to a cognitive behavioral approach, using a randomized controlled trial. We hypothesized a significant improvement in self-esteem after 10 weekly sessions of treatment. It was hypothesized that the results associated with both interventions would be maintained at 3 months follow-up. The second aim of the study was to examine the difference in effectiveness between both treatments.

## Materials and methods

### Design

The protocol of the study was approved by the Medical Ethics Committee (NL49421.044.14) and was registered on May 27th, 2014 (www.trialregister.nl) with identifier NTR4611. It used a randomized controlled trial (1:1 allocation ratio) with two parallel groups, i.e., an EMDR condition and a CBT condition. Randomization was executed (with concealment of allocation) through central randomization performed by an independent randomizer (http://www.randomizer.org) using random assignment with “a two blocked design” (to keep sample size equal across conditions) in order of date of entry of the study.

### Participants

**Figure 1:**
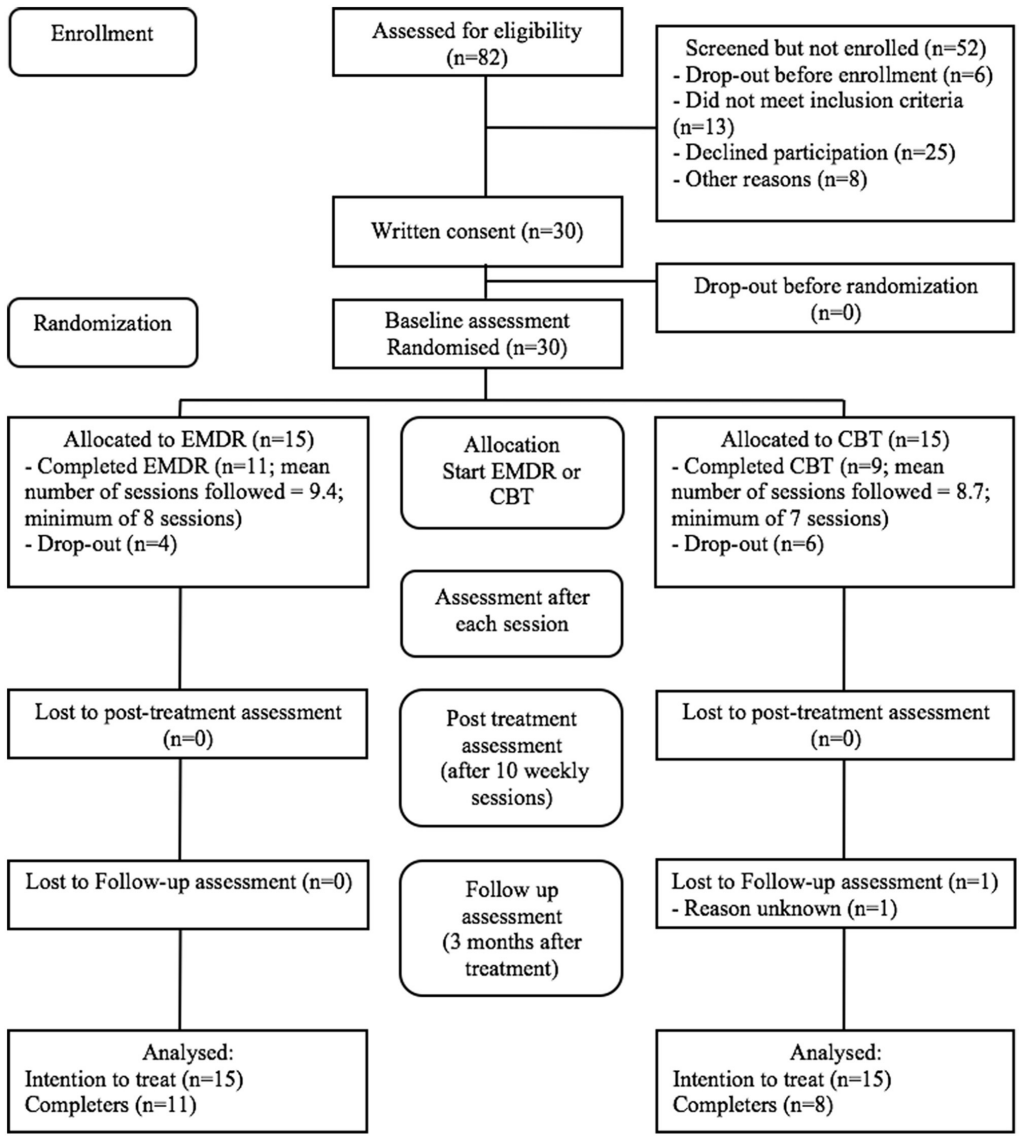
Flow chart of the trial.

The study participants were recruited at a health care center for secondary mental health. During the study period (i.e., from October 2014 through July 2016), a total of 82 patients were referred for self-esteem treatment and were informed about the study. Thirty patients met the inclusion criteria and were willing to participate. They were included and randomized to either EMDR therapy (*n* = 15) or CBT (*n* = 15). Figure 1 shows the flow of patients through the study. During the study 10 patients (four in the EMDR and six in the CBT condition) dropped out for various reasons, for example due to a sudden loss in the family, acute suicidality before starting treatment, a preference for a certain treatment condition while not being included in that condition, or wanting to follow other treatments for more prominent disorders. Ultimately, 20 patients underwent the full treatment protocol, i.e., 11 patients in the EMDR condition and nine in the CBT condition. One patient in the CBT condition was lost to follow-up. Baseline characteristics of the sample are shown in Table 1.

**Table 1:** Demographic and Diagnostic Characteristics of Intention-to-Treat and Treatment Completer Samples, divided by group allocation.

**Variable**	**Intention-to-Treat**		**Completers**	
	**EMDR**	**CBT**	**EMDR**	**CBT**
	**(*n* = 15)**	**(*n* = 15)**	**(*n* = 11)**	**(*n* = 8)**
Mean age	38,8	28,6	41,5	32,1
**SEX**				
Male	2	3	2	2
Female	13	12	9	6
**MOOD DISORDER**				
Depressive disorder	7	5	6	2
Dysthymic disorder	5	4	4	1
**ANXIETY DISORDER**				
Social phobia	1	2	1	2
Specific phobia	1	–	1	–
Panic disorder with agoraphobia	1	2	1	1
Panic disorder without agoraphobia	1	–	1	–
Agoraphobia without history of panic disorder	1	–	–	–
Generalized anxiety disorder	3	3	3	–
Obsessive compulsive disorder	–	1	–	1
Anxiety disorder NOS	2	–	1	–
**DEVELOPMENTAL DISORDER**				
Autistic spectrum disorder	–	2	–	1
ADHD	3	3	2	1
**SOMATOFORM DISORDER**				
Undifferentiated somatoform disorder	–	1	–	1
**EATING DISORDER**				
Eating disorder NOS	1	2	1	–
**SUBSTANCE RELATED DISORDERS**				
Alcohol dependence	1	1	–	1
Cannabis dependence	–	1	–	–
Sedative-, hypnotic-, or anxiolytic related disorder	1	–	–	–
**ADDITIONAL CODES**				
Partner relational problem	–	1	–	1
Identity problem	1	–	1	–
Psychological factors affecting medical condition	1	–	1	–
**PERSONALITY DISORDER**				
Borderline personality disorder	3	3	1	1
Avoidant personality disorder	2	1	2	1
Personality disorder NOS	4	3	3	2
Personality disorder deferred	5	5	4	3
No diagnosis on Axis II	2	2	2	1
**CO-MORBIDITY**				
Multiple Axis I diagnosis	11	10	9	4
Axis I and Axis II diagnosis or deferred	13	13	9	7
Multiple Axis II diagnosis or deferred	1	–	1	–

*Diagnosis according to the Diagnostic Statistical Manual-IV-TR (DSM-IV-TR)*.

The inclusion criteria of the study were an age between 18 and 65 years, a reference by their therapist for the treatment of their self-esteem, having a low self-esteem as indexed by a score below the cut-off point (<16) on the Rosenberg Self-esteem Scale, having an Axis I and/or Axis II disorder according to the DSM-IV-TR (American Psychiatric Association, 2000) diagnosed by their referring therapist, other than a PTSD, sufficient mastery of the Dutch language, and being capable of doing homework. During the study period patients were not allowed to receive other treatments.

### Procedure

The study participants, already diagnosed with an Axis I and/or II disorder, were referred for self-esteem treatment by their mental health professional. They were screened for low self-esteem with the Rosenberg Self-esteem Scale (RSES) and assessed for PTSD with the MINI-International Neuropsychiatric Interview (Van Vliet and De Beurs, 2007). When patients met the inclusion criteria they were informed about the study, verbally and in writing. One week later, one of the researchers had telephone contact about participating, answered possible questions and formally invited the patient to participate. After the informed consent form was signed, the baseline assessment and randomization to the EMDR or CBT condition took place. Patients were assessed at baseline (T0) regarding self-esteem (RSES and Credibility of Core Beliefs), psychological symptoms (Brief Symptom Inventory) and both social anxiety and social interaction IIS. Before treatment, the negative core belief that was most representative of patients' low self-esteem was selected using the “Downward arrow technique” (Beck, 1995). In contrast, a positive alternative belief was formulated by the patient in reaction to the question as to what they would rather believe instead of their negative core belief. The affective credibility of the beliefs was scored on a Visual Analogue Scale (VAS) ranging from 0 to 100% credibility (Credibility of Core Beliefs). After each of the 10 treatment sessions, patients were assessed with the Credibility of Core Beliefs and with the Rosenberg Self-esteem Scale. After 10 weeks of treatment (T1), and at 3 months follow-up (T2) all patients were assessed again on all the outcome measures.

### Assessment measures

It was hypothesized that the treatments would enhance self-esteem, reduce psychiatric symptoms in general, reduce social anxiety, and would increase social interaction.

#### Rosenberg self-esteem scale

The RSES was used as primary outcome measure for self-esteem. This widely used questionnaire (Schmitt and Allik, 2005) is a 10-item self-report measure to assess global self-esteem by asking the respondents to reflect on their current feelings on a four-point scale (0 = “strongly disagree” 3 = “strongly agree”; Rosenberg, 1965; Franck et al., 2008). Total scores range from 0 up to 30, with higher scores indicating a higher global self-esteem. The cut-off for inclusion was 16, so that participants at baseline all scored at least 1 standard deviation (*SD* = 4) below the mean of 20 (Franck et al., 2008). The Dutch version of the RSES has good internal consistency and test–retest reliability (Everaert et al., 2010).

#### Credibility of core beliefs

The affective credibility of the negative core belief (CNCB) and the credibility of the positive alternative belief (CPAB) were scored on a visual analog scale ranging from 0 to 100% credibility.

#### Brief symptom inventory

The Brief Symptom Inventory (BSI) is an abbreviated version of the SCL-90-R questionnaire, consisting of 53-items, and is an index for severity of psychological symptoms (Derogatis and Melisaratos, 1983). The BSI rates the extent to which individuals have been bothered (0 = “not at all” to 4 = “extremely”) in the past week by various symptoms. In the present study the BSI Total Score was used as outcome measure which represents the overall degree of mental illness. The reliability of the Total Score is sufficient and the discriminant validity of the Dutch version is good (De Beurs and Zitman, 2006).

#### Inventory of interpersonal situations

The Inventory of Interpersonal Situations (IIS) is a Dutch self-report questionnaire measuring social anxiety and social interaction (Van Dam-Baggen and Kraaimaat, 2004). The questionnaire consists of two parts, the first part determining the extent to which discomfort is experienced in certain social situations and the second part determining the frequency of the social interaction. The questionnaire consists of 35 items ranging from 1 to 5 (part 1; 1 = “not at all” 5 = “very much”, part 2; 1 = “never” 5 = “always”). Several studies support the high predictive validity and the reliability of the IIS Discomfort and Frequency scales (Van Dam-Baggen and Kraaimaat, 1999).

### Treatments

#### EMDR condition

Patients received 10 weekly sessions of 75 min each. For the case conceptualizations addressing patients' self-esteem the “second method” was used (De Jongh et al., 2010). The underlying principle of this method of case conceptualization is that negative events leave memory traces causing and maintaining dysfunctional core beliefs. According to this method, five of the most relevant memories that contributed to the formation and the present credibility of the selected negative core belief were identified. More specifically, in the present study the patient was requested to select the memories that subjectively “proved” that the belief was true and to describe the content of these memories in a few sentences. EMDR therapy, using the Standard protocol (De Jongh and Ten Broeke, 2003), started with the memory which, according to the patient, was considered providing the strongest “proof” for the negative core belief; that is, the memory associated with the dysfunctional meaning (e.g., “I'm worthless”). Next, a more functional belief about the person (e.g., “I'm okay”; Shapiro, 2002) was installed. When the memory was effectively treated, meaning the Subjective Units of Distress scale (SUD) reported by participants was at least 2 or lower (range 0–10), the next memory that provided the most evidence for the negative core belief was selected and processed. This was repeated for the other memories.

#### CBT condition

Patients received 10 weekly group sessions of CBT of 120 min each including a 15 min break. The CBT group, consisting of 6 to 10 patients, was based on the “Whitebook Method” described by De Neef (2010) that uses “positive data logging” (Padesky, 1994) to specifically focus on evidence that is contradictory to the negative core belief. Patients received psycho-education about how information that is contradictory to the negative core belief is usually discounted and distorted leading to not noticing and evaluating exceptions to their negative core belief. Patients kept a positive data log to write down positive events and positive qualities of themselves. Additionally they investigated pro's and cons of negative thoughts, received information and training about receiving criticism and they discussed how to prevent relapse.

### Treatment integrity

All EMDR and CBT sessions were videotaped. Feedback on adherence to the EMDR or CBT protocol and the competence of the therapists was given by licensed EMDR or CBT supervisors to optimize the quality and equality of the treatments. Case conceptualizations of each patient in the EMDR condition were checked and evaluated with the therapists by two EMDR supervisors before commencing treatment. The EMDR therapists were trained to perform EMDR for low self-esteem, using the “Second method,” whereas the group therapists received extensive general training in CBT and were qualified to perform the CBT protocol for low self-esteem as described by De Neef (2010).

### Statistical analysis

All analyses were conducted with SPSS for Windows version 23.0. Independent samples *t*-tests and Chi-square tests were performed to analyse differences between treatment conditions pre-treatment. This was done for both the intention-to-treat sample (*n* = 30) and the complete cases (*n* = 19), i.e., patients who finished the whole research protocol. For the variables that were not normally distributed, the Mann-Whitney U test was used. In the Chi-square analyses the Yates' correction was used (Yates, 1934) to prevent overestimation of statistical significance for small groups. Using descriptive statistics, the scores on the self-esteem measures over the course of sessions (RSES and CCB) were explored.

A repeated measures ANOVA was performed for each of the outcome variables on all complete cases (patients who completed the full research protocol) to examine the effect of treatment condition on self-esteem, psychological symptoms, social anxiety, and frequency of social interaction (GLM: general linear model, repeated measures). Time (pre-treatment, post treatment and follow-up) was used as a within-subject variable and treatment (EMDR vs. CBT) as a between-subject variable. To determine to what extent patients showed improvement over time a Helmert contrast was used to directly compare pre-treatment (T0) to post treatment (T1) and follow-up (T2) and post treatment (T1) with follow-up (T2). Not all variables were normally distributed but ANOVA is considered fairly robust to such a violation (Stevens, 2002). Since the assumption of sphericity was violated in most of the variables (Mauchly's Test of Sphericity *p* < 0.05), the Greenhouse Geisser correction was applied. For all comparisons effect sizes were calculated (small effect: $\eta_{p}^{2}$ = 0.01; medium effect: $\eta_{p}^{2}$ = 0.06; large effect: $\eta_{p}^{2}$ = 0.14) (Fritz et al., 2012). Furthermore, an intention-to-treat analysis was performed, using the last observation carried forward method, and a non-parametric analysis, using the Friedman test, was performed to examine the robustness of the ANOVA results in the complete cases.

A reliable change (RC) index was calculated to determine which patients' RSES, BSI, and IIS scores changed beyond a level that could be attributed to measurement error (Evans et al., 1998). For this purpose, the standard error of measurement of the difference (SEdiff) was used, which takes account of the 2 measurements (pre-treatment and post treatment). The formula is $SE_{diff}=\;SD_{1}\sqrt{2}\sqrt{1-\alpha }$, where SD1 is the standard deviation of the baseline observations and alpha is the reliability of the measure (Cronbach alpha coefficient). It is assumed that change that exceeds 1.96 times this standard error (i.e., the RC index) is unlikely to occur more than 5% of the time by unreliability of the measure alone (Evans et al., 1998). In addition, a clinical significant change criterion was calculated to determine which patients' RSES, BSI, and IIS scores changed to a level that could be considered clinically meaningful. The cut-off point was determined according to “criterion C,” i.e., where the likelihood of the patient being in the normative distribution was greater than being in the clinical distribution after treatment (Evans et al., 1998). The cut-off point was set at where the SD's of the clinical and normative data were equal: $\frac{\left(mean_{clin}\;\times \;SD_{norm}\right)+\left(mean_{norm}\;\times \;SD_{clin\;}\right)}{SD_{norm}+SD_{clin}}$ (Evans et al., 1998).

## Results

### Participants and randomization

Considering the demographic characteristics (intention-to-treat), there was a significant age difference between the two treatment conditions [*t*(28) = 2.81, *p* = 0.01], the mean age of the EMDR condition being significantly higher (*M* = 38.8, *SD* = 11.83) than in the CBT condition (*M* = 28.6, *SD* = 7.64). The sex ratio in sample did not differ from expectation [Chi-square = 0.21 (1), *p* = 0.65]. As for diagnoses, no significant differences between groups were found, with the only exception that the prevalence of mood disorders within the complete cases was significantly [Chi-square = 7.21 (1), *p* = 0.01] higher in the EMDR condition (10) than in the CBT condition (3). For the baseline measures of all the outcome variables there were no significant pre-experimental differences in scores measuring self-esteem, psychological symptoms, social anxiety, and social interaction between the EMDR and the CBT condition. This was the case for the intention-to-treat as well as the complete cases.

### Treatment participation

No significant between-group difference in the number of sessions that were completed was found [*t* = 1.42(28), *p* = 0.17]. For the complete cases, patients in the EMDR condition completed at least 8 of the 10 sessions (*M* = 9.36, *SD* = 0.81), whereas in the CBT at least seven sessions of the 10 sessions were completed (*M* = 8.67, *SD* = 1.32). In the EMDR condition, the mean of the SUD scores of the selected targets before desensitization was 7.6 (scale 0–10). In the EMDR condition, a mean of 4 memories were reprocessed to a SUD score of 2 or lower.

### Changes in self-esteem over sessions

As to the scores on the CNCB over the sessions, the mean scores of the patients in the EMDR condition dropped below 50% credibility in session #7 and this was maintained throughout session #8, #9, and #10. Looking at individual scores, more than half of the patients in the EMDR condition (6 patients) dropped below 50% credibility in session #5. For the CBT condition the mean score on CNCB dropped below 50% credibility, being more not true than true, in session #8 and this was maintained in session #9 and #10. Also in session #8, more than half of the patients in the CBT condition (5 patients) reached an individual score below 50% credibility.

**Figure 2:**
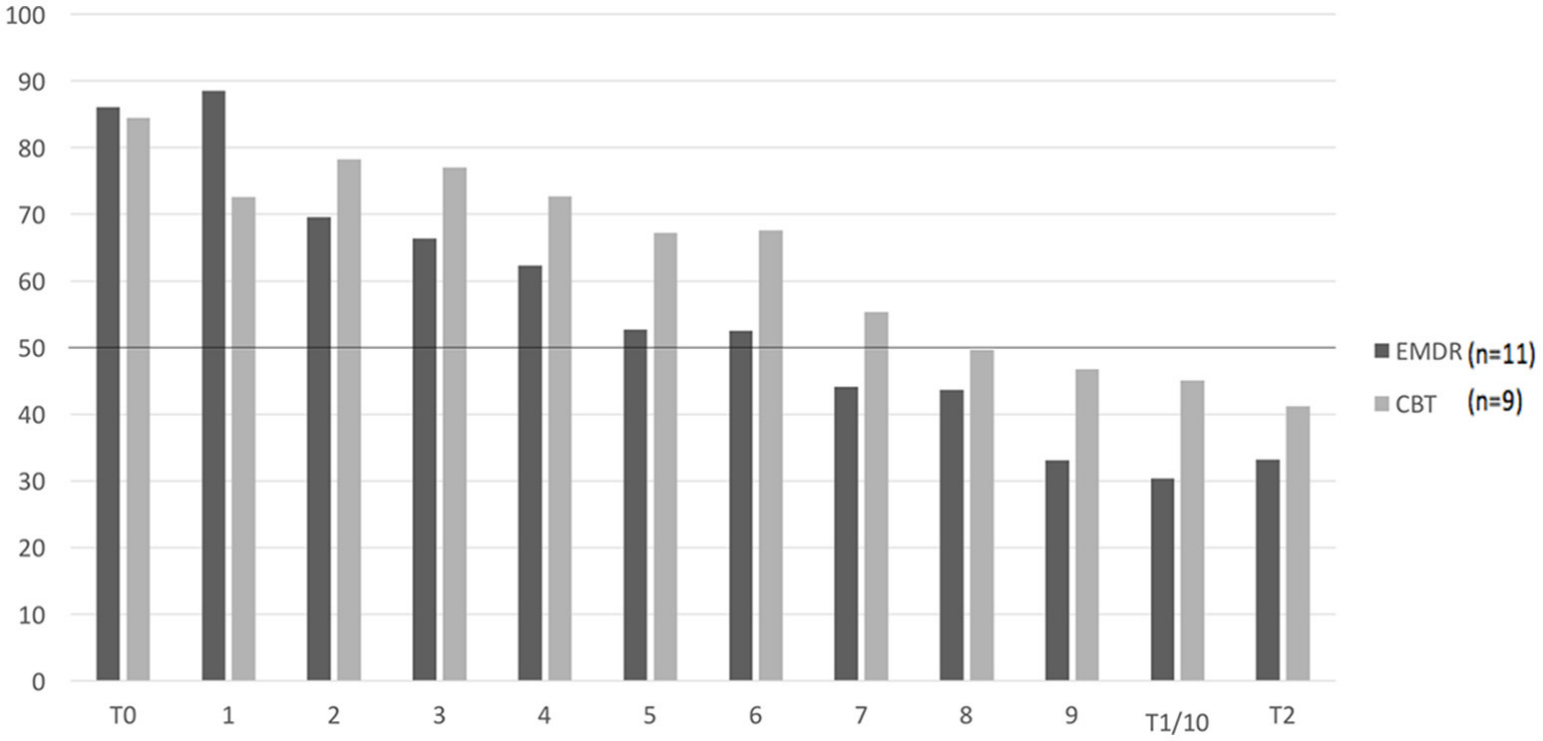
Mean scores on the CNCB per condition over the course of treatment (*n* = 20). CNCB, Credibility of Negative Core Belief. T0: pre-treatment, T1: post-treatment, T3: months follow-up. 1–10: weekly sessions. 0–100%: credibility of core belief. Missing values were imputed with last observation carried forward.

**Figure 3:**
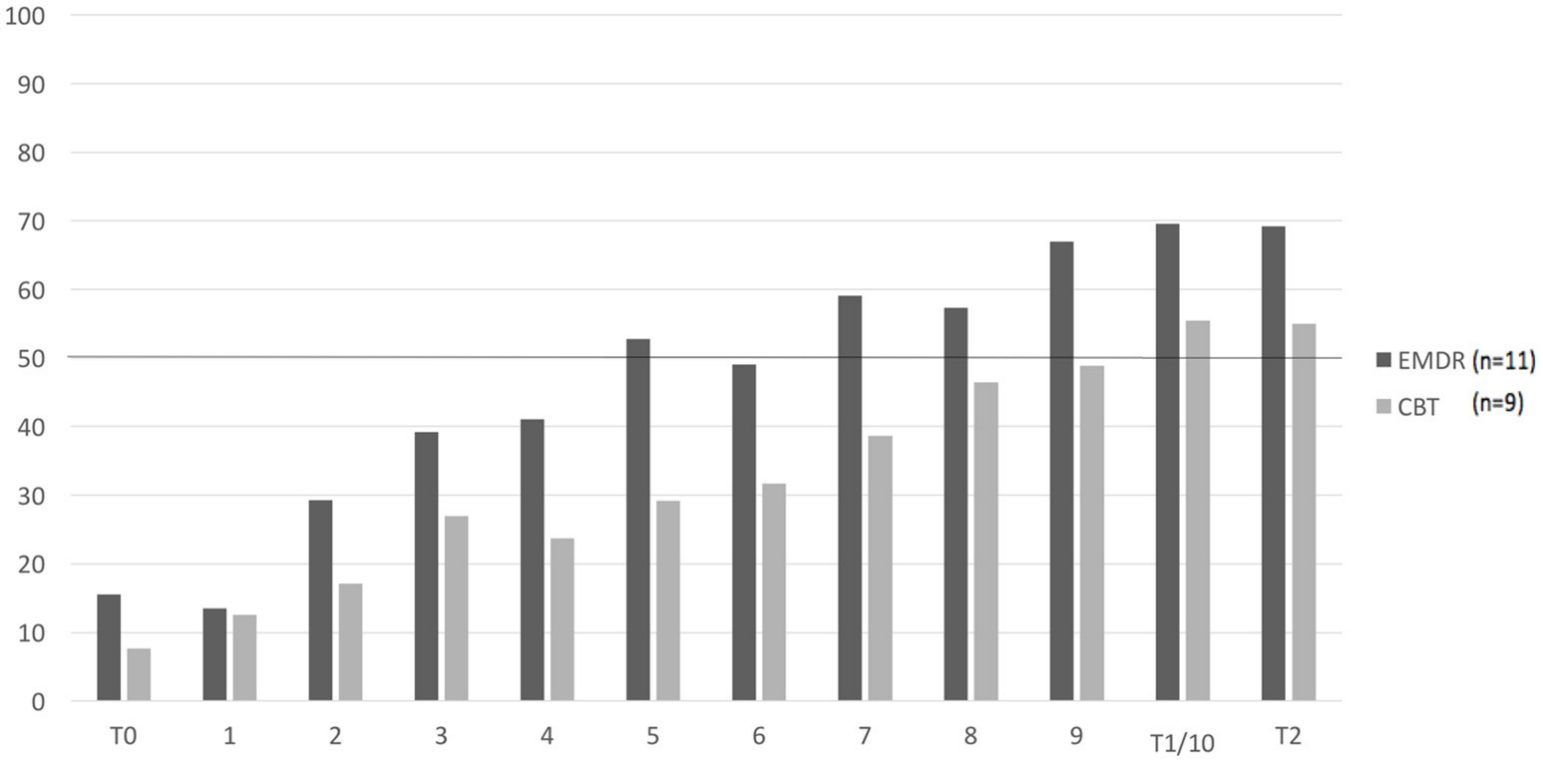
Mean scores on the CPAB per condition over the course of treatment (*n* = 20). CPAB, Credibility of Positive Alternative Belief. T0: pre-treatment, T1: post-treatment, T3: months follow-up. 1–10: weekly sessions. 0–100%: credibility of core belief. Missing values were imputed with last observation carried forward.

For the positive alternative belief, credibility exceeded 50% credibility in session #7 for the EMDR and in session #10 in the CBT condition. More than half of the patients in each group exceeded 50% credibility in session #5 for the EMDR and in session #9 for the CBT condition. Figures 2 and 3 show the mean scores on the CNCB and the positive alternative belief per group over the course of the treatment.

**Figure 4:**
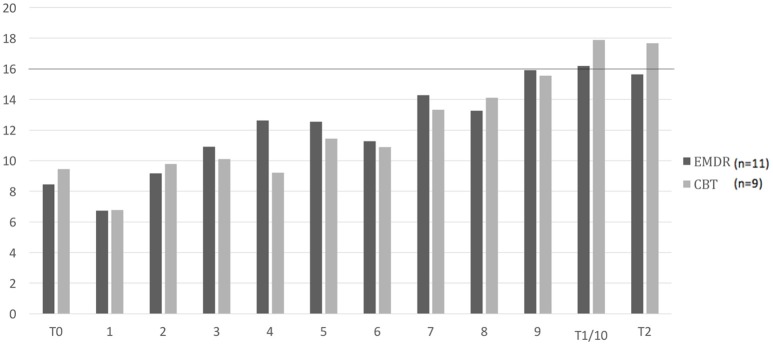
Mean scores on the RSES per condition over the course of treatment (*n* = 20). RSES, Rosenberg Self-esteem Scale. T0: pre-treatment, T1: post-treatment, T3: months follow-up. 1–10: weekly sessions. Cut-off score 16. Missing values were imputed with last observation carried forward.

When looking at the scores on the RSES over the sessions, the mean of the patients in the EMDR condition reached a score of 16 (cut-off) or higher in session #9. This was also the case in session #9 in the CBT condition. More than half of the patients reached a score of 16 or higher in session #9 in the EMDR condition, this was in session #10 for the CBT condition. Figure 4 shows the mean scores on the RSES per group over the course of treatment.

### Treatment effects

Table 2 displays the means and standard deviations for the various outcome measures, measurement times, and therapy types. The ANOVA analysis for the complete cases showed a significant improvement over time on all the outcome measures as shown in Table 3. Regarding all measures the interaction between time and treatment condition was, however, not significant, congruently showing very small effect sizes. This indicates that there were no significant differences between the EMDR and CBT condition on any of the measures. Yet, significant increases of self-esteem and social interaction as well as decreases of psychological symptoms and social anxiety were seen for both treatment conditions. The Friedman test yielded similar results for the self-esteem measures and the measure for psychological symptoms except for social anxiety, whereas social interaction significantly increased over time in the CBT condition, but not in the EMDR condition. The intention-to-treat analysis showed significant improvements that for all outcome measures from pre-treatment (T0) compared to post-treatment (T1) and follow-up (T2). For the complete cases, no differences were found between T1 and T2, indicating that the treatment results that were achieved in both the EMDR and CBT condition between T0 and T1 were maintained at T2. The intention-to-treat analysis showed similar results. For more detailed information on the intention-to-treat sample, we refer to Table A1 in Appendix.

**Table 2:** Means (SD) of the outcome measures.

	**Condition 1; EMDR**			**Condition 2; CBT**		
	**T0 (*n* = 11)**	**T1 (*n* = 11)**	**T2 (*n* = 11)**	**T0 (*n* = 8)**	**T1 (*n* = 8)**	**T2 (*n* = 8)**
RSES	8.45	16.18	15.64	9.00	18.13	17.88
	(4.44)	(10.17)	(9.09)	(3.51)	(7.24)	(8.37)
CNCB	86.09	30.36	33.18	90.75	47.50	43.12
	(17.46)	(37.42)	(37.99)	(7.78)	(32.20)	(36.52)
CPAB	15.55	69.55	69.18	7.75	57.38	56.88
	(19.31)	(36.97)	(36.06)	(6.16)	(33.49)	(33.27)
BSI	1.73	1.27	1.17	1.78	1.09	1.13
	(1.03)	(1.19)	(1.16)	(0.95)	(0.70)	(0.83)
IIS DISC	112.45	95.18	88.91	110.63	86.00	83.63
	(31.83)	(39.73)	(38.37)	(25.43)	(26.40)	(24.85)
IIS FREQ	82.73	92.18	95.27	85.13	100.63	109.38
	(11.47)	(30.06)	(27.55)	(17.72)	(22.52)	(19.98)

*RSES, Rosenberg Self-esteem Scale; CNCB, Credibility of Negative Core Belief; CPAB, Credibility of Positive Alternative Belief; BSI, Brief Symptom Inventory; IIS DISC, Inventory of Interpersonal Situations, Discomfort in Social Interactions; IIS FREQ, Inventory of Interpersonal Situations, Frequency of Social interaction; T0, Pre-treatment; T1, Post treatment; T2, 3 months follow-up*.

**Table 3:** ANOVA analysis for the complete cases (*n* = 19).

	**Effect Time**			**Effect time** × **condition**			**T0 vs. T1 and T2**			**T1 vs. T2**		
	***F***	***P***	$\eta_{p}^{2}$	***F***	***p***	$\eta_{p}^{2}$	***F***	***p***	$\eta_{p}^{2}$	***F***	***p***	$\eta_{p}^{2}$
RSES	16.30	0.00	0.49	0.15	0.77	0.01	18.80	0.00	0.53	0.21	0.65	0.01
CNCB	28.56	0.00	0.63	0.34	0.59	0.02	29.92	0.00	0.64	0.12	0.74	0.01
CPAB	36.30	0.00	0.68	0.07	0.81	0.00	37.54	0.00	0.69	0.06	0.81	0.00
BSI	10.51	0.00	0.38	0.29	0.68	0.02	13.45	0.00	0.44	0.10	0.76	0.01
IIS DISC	10.40	0.00	0.38	0.19	0.76	0.01	12.75	0.00	0.43	1.30	0.27	0.07
IIS FREQ	5.74	0.01	0.25	0.56	0.55	0.03	7.59	0.01	0.31	1.79	0.20	0.10

*RSES, Rosenberg Self-esteem Scale; CNCB, Credibility of Negative Core Belief; CPAB, Credibility of Positive Alternative Belief; BSI, Brief Symptom Inventory; IIS DISC, Inventory of Interpersonal situations; Discomfort in Social Interactions, IIS FREQ, Inventory of Interpersonal Situations; Frequency of social interaction; T0, Pre-treatment; T1, Post treatment; T2, 3 months follow-up*.

### Reliable and clinical change

The self-esteem measure (RSES) showed the highest percentage clinically relevant change (60%), followed by social anxiety (40%), social interaction (35%), and finally psychological symptoms (25%). For the specific percentages in the different treatment groups, see Table 4.

**Table 4:** Percentage of patients showing reliable and clinical significant changes on self-esteem, psychological symptoms and social interaction (*n* = 20).

		**Total group (*****n*** = **20) reliable change**		**EMDR (*****n*** = **11)**		**CBT (*****n*** = **9)**	
		**Yes**	**No**	**Yes**	**No**	**Yes**	**No**
		**(%)**	**(%)**	**(%)**	**(%)**	**(%)**	**(%)**
**CLINICAL CHANGE**							
RSES (>14)	60%						
		55	5	55	0	56	11
BSI (<0.80)	25%						
		15	10	27	0	0	22
IIS DISC (<86)	40%						
		30	10	36	9	22	11
IIS FREQ (>95)	35%						
		35	0	27	0	44	0

*RSES, Rosenberg Self-esteem Scale; BSI, Brief Symptom Inventory; IIS DISC, Inventory of Interpersonal Situations; Discomfort in Social Interaction; IIS FREQ, Inventory of Interpersonal Situations, Frequency of Social Interaction*.

## Discussion

The results of the present study suggest that both EMDR therapy and CBT have the potential to be an effective treatment alternative for patients who suffer from low self-esteem in co-occurrence with a wide range of psychiatric disorders.

Patients improved not only more than two standard deviations on the primary outcome measure (Rosenberg Self-esteem Scale), the treatments also led to significant reductions in general psychiatric symptoms and social anxiety, as well as to a significant increase of social interactions. All treatment effects were maintained at 3 months follow up. These results were held after an intention-to-treat analysis was performed that included all patients who dropped out early in treatment. For the majority of the patients (60%), the amount of 10 therapy sessions resulted in a clinically significant improvement in self-esteem. No significant differences could be detected between the two therapies.

The results of this study are in line with the study of Wanders et al. (2008) who found similar effects in adolescents, in that EMDR therapy and CBT proved equally effective in changing low self-esteem. Conversely, the results are at odds with those of Staring et al. (2016) who found EMDR to be less effective in treating low self-esteem than COMET. Patients in the current study showed a larger improvement on self-esteem compared to Staring et al. (2016). This difference in results may be explained by the amount of sessions provided, in that Staring et al. (2016) used six sessions whereas the patients in the current study received ten sessions. Also it is likely that the memories targeted with EMDR in the current study with patients with multiple psychiatric diagnoses, were more emotionally charged and hence more susceptible for processing using EMDR therapy (Littel et al., 2017). Concerning CBT, in contrast to Ritter et al. (2013), who used 25 sessions of CBT to treat low self-esteem, we found that 10 sessions of CBT were sufficient to establish changes in self–esteem in the majority of the patients. The effectiveness of CBT in changing low self-esteem found in the present study (effect size on the RSES $\eta_{p\;}^{2}=$ 0.49), is in line with former studies on group CBT (Rigby and Waite, 2006; Morton et al., 2012; Pack and Condren, 2014).

This study had several strengths. Firstly, it is one of the first RCTs explicitly focussed on the effectiveness of EMDR therapy for low self-esteem in adults, and also one of the first RCTs examining the efficacy of CBT in treating low self-esteem. In contrast to former studies examining the effect of EMDR on low self-esteem (Wanders et al., 2008; Staring et al., 2016), the current study explicitly excluded patients with PTSD, making it more likely that the EMDR therapy was in fact effective in changing self-esteem instead of treating trauma related symptomatology. Secondly, regarding self-esteem treatment, the present study was one of the first to include a diverse patient group with various psychiatric disorders. The results suggest that EMDR as well as CBT are effective for treating low self-esteem in such a difficult population. Finally, this study used a follow-up measure to examine the treatment outcomes over time, showing that the treatment effects of both EMDR and CBT were maintained.

While the present study results are encouraging, there are a number of limitations that need to be acknowledged. First, given the relatively small sample size, it cannot be ruled out that the fact that no differences between groups were found were due to the fact that this study was underpowered. Secondly, because the EMDR treatment was delivered individually whereas the CBT treatment was given in a group setting, it could be argued that the experience of being accepted within a group and meeting other people who share similar difficulties, would be therapeutic for individuals with low self-esteem. Conversely, patients in the EMDR condition could have profited more from the individual attention of the therapist, feeling perhaps more comfortable in this context to display their deepest feelings and beliefs. Thirdly, there was a significant difference in age between patients in the EMDR and CBT condition. However, age differences in self-esteem generally appears to be relatively small compared to interindividual differences, like personality traits, and measurement error (Pullman et al., 2009; Orth et al., 2010). This is in line with the pre-treatment measurements as found in the current study in that despite the difference in age between both groups differences on self-esteem measures were lacking. Finally, this study lacked a passive control group, so it cannot be ruled out, however unlikely, that patients improved simply because of getting attention from the therapist and not because of the specific treatments methods.

Looking at an individual level, not all patients benefited equally from treatment. This was the case for the CBT as well as for the EMDR condition. Given that both treatments were effective at group level, specific patient groups might have benefited more or less from different kinds of interventions. Likewise, while for the majority of the patients ten sessions were enough to reach a clinical significant improvement in self-esteem, for the non-responders perhaps more sessions may have been needed, or perhaps they would have benefited more from another treatment method. The fact that no significant differences were found between groups does not support the hypothesis that EMDR might intervene on a more affective level than CBT. However, the results of this study indicate that EMDR can be used as an effective alternative for CBT in treating low self-esteem. Further research is warranted to examine whether certain patient groups might benefit more from one or the other treatment method, or a combination of both.

In conclusion, the present study is the first RCT examining the effectiveness of EMDR therapy and CBT on treating low self-esteem in a general psychiatric, adult, population. Despite the small sample size, the results suggests that, when using 10 sessions, both therapies seem effective for treating low self-esteem in patients with a wide range of psychiatric disorders in secondary mental health care. Future research will be needed to examine whether these findings can be replicated in a larger patient group, preferably using a waiting list control group. Furthermore, future studies should aim at examining which method for treating self-esteem works best for whom.

## Ethics statement

This study was carried out in accordance with the recommendations of the Medical Ethics Committee Twente with written informed consent from all subjects. All subjects gave written informed consent in accordance with the Declaration of Helsinki. The protocol was approved by the Medical Ethics Committee Twente.

## Author contributions

BG and AvdV designed the study, collected the data and wrote the manuscript; BG performed the data analysis; IdG and AdJ made substantial contributions to the conception and design of the study and edited and revised the manuscript. All authors read and approved the final manuscript.

### Conflict of interest statement

The authors declare that the research was conducted in the absence of any commercial or financial relationships that could be construed as a potential conflict of interest. AdJ receives income for published books on EMDR therapy and for the training of postdoctoral professionals in this method.

## Appendix

**Table A1:** Means (SD) of the outcome measures of intention to treat sample.

	**Condition 1; EMDR**			**Condition 2; CBT**		
	**T0 (*n* = 15)**	**T1 (*n* = 15)**	**T2 (*n* = 15)**	**T0 (*n* = 15)**	**T1 (*n* = 15)**	**T2 (*n* = 15)**
RSES	9.33	15.07	14.67	8.00	12.93	12.67
	(4.25)	(8.92)	(8.00)	(4.38)	(8.70)	(8.92)
CNCB	87.07	41.47	42.93	87.93	63.73	62.67
	(15.31)	(41.27)	(41.59)	(17.41)	(34.23)	(37.88)
CPAB	16.60	60.80	60.60	7.13	37.87	35.60
	(18.80)	(39.84)	(39.23)	(6.29)	(35.21)	(34.65)
BSI	1.77	1.39	1.33	1.91	1.52	1.61
	(0.90)	(1.09)	(1.07)	(0.86)	(0.91)	(1.02)
IIS DISC	106.27	94.00	88.80	109.67	96.87	96.40
	(30.64)	(34.89)	(34.11)	(26.32)	(31.93)	(33.68)
IIS FREQ	87.07	94.73	95.80	88.47	96.80	101.33
	(12.46)	(26.14)	(23.40)	(16.73)	(20.19)	(20.52)

*RSES, Rosenberg Self-esteem Scale; CNCB, Credibility of Negative Core Belief; CPAB, Credibility of Positive Alternative Belief; BSI, Brief Symptom Inventory; IIS DISC, Inventory of Interpersonal Situations, Discomfort in social interactions; IIS FREQ, Inventory of Interpersonal Situations, Frequency of social interaction; T0, Pre-treatment; T1, Post treatment; T2, 3 months follow-up*.

## References

[B1] American Psychiatric Association (2000). Diagnostic and Statistical Manual of Mental Disorders, 4th Edn. Washington, DC: American Psychiatric Publishing.

[B2] Barrowclough C. Tarrier N. Humphreys L. Ward J. Gregg L. Andrews B. (2003). Self-esteem, in schizophrenia: relationships between self-evaluation, family attitudes, and symptomatology. J. Abnorm. Psychol. 112, 92–99. 10.1037/0021-843X.112.1.92 12653417

[B3] Beck J. S. (1995). Cognitive Therapy: Basics and Beyond. New York, NY: Guilford Press.

[B4] Brown G. W. Bifulco A. Andrews B. (1990). Self-esteem and depression. III. Aetiological issues. Soc. Psychiatry Psychiatr. Epidemiol. 25, 235–243. 10.1007/BF00788644 2237604

[B5] De Beurs E. Zitman F. G. (2006). De Brief Symptom Inventory (BSI). De betrouwbaarheid en validiteit van een handzaam alternatief voor de SCL-90. (The brief Symptom Inventory (BSI): the reliability and validity of a brief alternative of the SCL-90). Maandblad Geestelijke Volksgezondheid 61, 120–141.

[B6] De Jongh A. Ten Broeke E. (2003). Handboek EMDR: Een Geprotocolleerde Behandelmethode Voor de Gevolgen van Psychotrauma (Handbook EMDR: A Protocolized Treatment Method for the Consequences of Psychotrauma), 6th Edn. Amsterdam: Pearson Assessment and Information B.V.

[B7] De Jongh A. Ten Broeke E. Meijer S. (2010). Two method approach: a case conceptualization model in the context of EMDR. J. EMDR Pract. Res. 4, 12–21. 10.1891/1933-3196.4.1.12

[B8] De Neef M. (2010). Negatief Zelfbeeld. Amsterdam: Uitgeverij Boom.

[B9] Derogatis L. R. Melisaratos N. (1983). The Brief Symptom Inventory: an introductory report. Psychol. Med. 13, 595–605. 10.1017/S0033291700048017 6622612

[B10] Dziegielewski S. Wolfe P. (2000). Eye Movement Desensitization and Reprocessing (EMDR) as a time limited treatment intervention for body image disturbance and self-esteem: a single subject case study design. J. Psychother. Independ. Pract. 1, 1–16. 10.1300/J288v01n03_01

[B11] Ehntholt K. A. Salkovskis P. M. Rimes K. A. (1999). Obsessive-compulsive disorder, anxiety disorders, and self-esteem: an exploratory study. Behav. Res. Ther. 37, 771–781. 10.1016/S0005-7967(98)00177-6 10452177

[B12] Evans C. Margison F. Barkham M. (1998). The contribution of reliable and clinically significant change methods to evidence-based mental health. Evid. Based Mental Health 1, 70–72. 10.1136/ebmh.1.3.70

[B13] Everaert J. Koster E. Schacht R. De Raedt R. (2010). Evaluatie van de psychometrische eigenschappen van de Rosenberg zelfwaardeschaal in een poliklinisch psychiatrische populatie. Gedragstherapie 43, 307–317. Available online at: http://doi.org/1854/LU-1100824

[B14] Fennell M. (1998). Cognitive therapy in the treatment of low self-esteem. Adv. Psychiatr. Treat. 4, 296–304. 10.1192/apt.4.5.296

[B15] Fennell M. (1999). Overcoming Low Self-Esteem. London: Constable & Robinson Ltd.

[B16] Franck E. De Raedt R. Barbez C. Rosseel Y. (2008). Psychometric properties of the Dutch Rosenberg self-esteem scale. Psychol. Belg. 48, 25–35. 10.5334/pb-48-1-25

[B17] Fritz C. O. Morris P. E. Richler J. J. (2012). Effect size estimates: Current use, calculations, and interpretation. J. Exp. Psychol. Gen. 141, 2–18. 10.1037/a0024338 21823805

[B18] Gual P. Perez-Gaspar M. Martinez-Gonzalez M. A. Lahortiga F. Irala-Estevez J. Cervera-Enguix S. (2002). Self-esteem, personality, and eating disorders: baseline assessment of a prospective population-based cohort. Int. J. Eat. Disord. 31, 261–273. 10.1002/eat.10040 11920987

[B19] Hewitt J. P. (2009). Oxford Handbook of Positive Psychology. Oxford, UK: Oxford.

[B20] Johnson S. Meyer B. Winnett C. Small J. (2000). Social support and self-esteem predict changes in bipolar depression but not mania. J. Affect. Disord. 58, 79–86. 10.1016/S0165-0327(99)00133-0 10760562

[B21] Littel M. Remijn M. Tinga A. M. Engelhard I. M. Van Den Hout M. (2017). Stress enhances the memory-degrading effects of eye movements on emotionally neutral memories. Clin. Psychol. Sci. 5, 316–324. 10.1177/2167702616687292

[B22] Lynum L. I. Wilberg T. Karterud S. (2008). Self-esteem in patients with borderline and avoidant personality disorders. Scand. J. Psychol. 49, 469–477. 10.1111/j.1467-9450.2008.00655.x 18564322

[B23] Maxwell J. P. (2003). The imprint of childhood physical and emotional abuse: a case study on the use of EMDR to address anxiety and a lack of self-esteem. J. Fam. Violence 18, 281–293. 10.1023/A:1025165227590

[B24] McManus F. Waite P. Shafran R. (2009). Cognitive-behavior therapy for low self- esteem: a case example. Cogn. Behav. Pract. 16, 266–275. 10.1016/j.cbpra.2008.12.007

[B25] Morton L. Roach L. Reid H. Stewart S. H. (2012). An evaluation of a CBT group for women with low self-esteem. Behav. Cogn. Psychother. 40, 221–225. 10.1017/S1352465811000294 21729343

[B26] Orth U. Trzesniewski K. H. Robins R. W. (2010). Self-esteem development from young adulthood to old age: a cohort-sequential longitudinal study. J. Pers. Soc. Psychol. 98, 645–658. 10.1037/a0018769 20307135

[B27] Pack S. Condren E. (2014). An evaluation of group cognitive behaviour therapy for low self-esteem in primary care. Cogn. Behav. Ther. 7, 1–10. 10.1017/S1754470X14000051

[B28] Padesky C. A. (1994). Schema change processes in cognitive therapy. Clin. Psychol. Psychother. 1, 267–278. 10.1002/cpp.5640010502

[B29] Parker T. J. Page A. C. Hooke G. R. (2013). The influence of individual, group, and relative self-esteem on outcome for patients undergoing group cognitive- behavioural therapy treatment. Br. J. Clin. Psychol. 52, 450 463. 10.1111/bjc.12029 24117916

[B30] Pullman H. Allik J. Realo A. (2009). Global Self-esteem across the life span: a cross-sectional comparison between representative and self-selected internet samples. Exp. Aging Res. 35, 20–44. 10.1080/03610730802544708 19173100

[B31] Rigby L. Waite S. (2006). Group therapy for self-esteem, using creative approaches and metaphor as clinical tools. Behav. Cogn. Psychother. 35, 361–364. 10.1017/S1352465806003389

[B32] Ritter V. Leichsenring F. Strauss B. Stangier U. (2013). Changes in implicit and explicit self-esteem following cognitive and psychodynamic therapy in social anxiety disorder. Psychother. Res. 23, 547–558. 10.1080/10503307.2013.802824 23742669

[B33] Rosenberg M. (1965). Society and the Adolescent Self-Image. Princeton, NJ: Princeton University Press.

[B34] Sanders D. Ten Broeke E. (2011). EMDR bij de behandeling van een negatief zelfbeeld. Psychopraktijk 3, 19–22. 10.1007/s13170-011-0039-z

[B35] Schmitt D. P. Allik J. (2005). Simultaneous administration of the Rosenberg self-esteem scale in 53 nations: exploring the universal and culture-specific features of global self-esteem. J. Pers. Soc. Psychol. 89, 623–642. 10.1037/0022-3514.89.4.623 16287423

[B36] Shapiro F. (2001). Eye Movement Desensitization and Reprocessing: Basic Principles, Protocols, and Procedures, 2nd Edn. New York, NY: Guilford Press.

[B37] Shapiro F. (ed.). (2002). EMDR as an Integrative Psychotherapy Approach: Experts of Diverse Orientations Explore the Paradigm Prism. Washington, DC: American Psychological Association.

[B38] Shapiro F. (2007). EMDR, adaptive information processing, and case conceptualization. J. EMDR Pract. Res. 1, 68–87. 10.1891/1933-3196.1.2.68

[B39] Silverstone P. H. Salsali M. (2003). Low self-esteem and psychiatric patients: part I- the relationship between low self-esteem and psychiatric diagnosis. Ann. Gen. Hosp. Psychiatry 2:2. 10.1186/1475-2832-2-2 12620127 PMC151271

[B40] Soares J. Grossi G. (2000). The relationship between levels of self-esteem, clinical variables, anxiety/depression and coping among patients with Musculoskeletal Pain. Scand. J. Occup. Ther. 7, 87–95. 10.1080/110381200750018887

[B41] Solomon R. M. Shapiro F. (2008). EMDR and the adaptive information processing model: potential mechanisms of change. J. EMDR Pract. Res. 2, 315–325. 10.1891/1933-3196.2.4.315

[B42] Sowislo J. F. Orth U. (2013). Does low self-esteem predict depression and anxiety? A meta-analysis of longitudinal studies. Psychol. Bull. 139, 213–240. 10.1037/a0028931 22730921

[B43] Staring A. B. P. van den Berg D. P. G. Cath D. C. Schoorl M. Engelhard I. M. Korrelboom C. W. (2016). Self-esteem treatment in anxiety: a randomized controlled crossover trial of Eye Movement Desensitization and Reprocessing (EMDR) versus Competitive Memory Training (COMET) in patients with anxiety disorders. Behav. Res. Ther. 82, 11–20. 10.1016/j.brat.2016.04.002 27155451

[B44] Stevens J. P. (2002). Applied Multivariate Statistics for the Social Sciences. Mahwah, NJ.

[B45] Van Dam-Baggen C. M. J. Kraaimaat F. W. (2004). Inventarisatielijst Omgaan met Anderen. Amsterdam: Pearson.

[B46] Van Dam-Baggen C. M. J. Kraaimaat F. (1999). Assessing social anxiety: the inventory of interpersonal situations (IIS). Eur. J. Psychol. Assess. 15, 25–38. 10.1027//1015-5759.15.1.25

[B47] Van Vliet I. M. De Beurs E. (2007). The mini-international neuropsychiatric interview. A brief structured diagnostic psychiatric interview for DSM-IV en ICD-10 psychiatric disorders. Tijdschrift Voor Psychiatrie 49, 393–397. 17614093

[B48] Waite P. McManus F. Shafran R. (2012). Cognitive behaviour therapy for low self-esteem: a preliminary randomized controlled trial in a primary care setting. J. Behav. Ther. Exp. Psychiatry 43, 1049–1057. 10.1016/j.jbtep.2012.04.006 22683442

[B49] Wanders F. Serra M. De Jongh A. (2008). EMDR versus CBT for children with self-esteem and behavioral problems: a randomized controlled trial. J. EMDR Pract. Res. 2, 180–189. 10.1891/1933-3196.2.3.180

[B50] Yates F. (1934). Contingency table involving small numbers and the χ2 test. Suppl. J. R. Stat. Soc. 1, 217–235. 10.2307/2983604

[B51] Young J. E. Zangwill W. M. Behary W. E. (2002). Combining EMDR and schema-focused therapy: the whole may be greater than the sum of the parts, in EMDR as an Integrative Psychotherapy Approach: Experts of Diverse Orientations Explore the Paradigm Prism, ed Shapiro F. (Washington, DC: American Psychological Association), 181–208.

